# Perception on Online Teaching and Learning Among Health Sciences Students in Higher Education Institutions during the COVID-19 Lockdown – Ways to Improve Teaching and Learning in Saudi Colleges and Universities

**DOI:** 10.12688/f1000research.28178.2

**Published:** 2021-12-07

**Authors:** Khalid Aziz Ansari, Faraz A. Farooqi, Soban Qadir Khan, Muhanad Alhareky, Ma. Abigail C. Trinidad, Taha Abidi, Muzaheed M

**Affiliations:** 1 Department of Respiratory Care, College of Applied Medical Sciences, Imam Abdulrahman Bin Faisal University, Damman, Saudi Arabia; 2 College of Dentistry, Imam Abdulrehman Bin Faisal University, Dammam, Dammam, Saudi Arabia; 3 Department of Preventive Dental Science, College of Dentistry, Imam Abdulrahman Bin Faisal University, Dammam, Saudi Arabia; 4 Department of Public Health, Saudi Electronic University, Riyadh, Saudi Arabia; 5 Department of Clinical Laboratory Science, College of Applied Medical Sciences, Imam Abdulrahman Bin Faisal University, Dammam, Saudi Arabia

**Keywords:** COVID-19, E-learning; Traditional learning; Student’s Perceptions

## Abstract

**Background: **Online learning or E-learning are approaches to broadcasting teaching by the means of internet technology and software applications. Kingdom of Saudi Arabia is likewise embarking on the rapid growth in online education. The purpose of this study is to investigate the student’s perceptions regarding online teaching and learning during COVID 19.

**Methods: **An online computer-based cross-sectional study was conducted between May and June 2020. A pre-validated questionnaire was used and administrated to health sciences students studying at Dammam Universities through online software QuestionPro.

**Results: **Out of total 281, 68% of the participants were females (n=188) while 31.9% (n=88) were male students with an average age of 23.1(4.5) years. Overall, 176 (62%) of the students expressed their satisfaction with online learning.

**Conclusion: **Findings will help academicians to identify strengths, areas of improvement, and encourage faculty to think deeply to restructure course learning objectives, teaching techniques to engage students and improve learning process.

## Introduction

In order to control the ongoing worsening situation of the spread of Coronavirus ^1^ amongst their residents, most countries declared a full or partial lockdown within their territories. ^2^ Various education ministries across the globe also took abrupt measures to minimize face-to-face contact among students and staff and advised higher education institutions to transform all academic teaching online. Therefore, all face-to-face academic activities were put on hold in the middle of the semester; and in order not to disrupt the provision of lectures in academic institutions such as colleges and universities, all academic activities were shifted towards virtual teaching. The virtual online educational system became a necessity and is now becoming popular as a normal teaching practice in various countries for the undefined future.

Online learning is a teaching method administered by means of the internet and software applications. ^3^ The rapid spread of this technique resulted in various universities actively targeting students worldwide to encourage them to attain an education online to save money. ^4^^–^^6^ The Kingdom of Saudi Arabia (KSA) is likewise embarking on the rapid growth in online education.

KSA is enhancing its educational goals and actively contributing to international educational changes to meet the challenges of future generations. ^7^^–^^8^ KSA has established several online universities such as Online Islamic University (2010) and The Saudi Electronic University (SEU) (2011). Citizens living far away from the main campuses and cities have to travel and live away from their families therefore online education will help them to stay in their hometown and still fulfill their educational desires. ^10^ In the KSA, there have been many attempts to blend online education with the traditional education system. However, no conclusive data is available on the students ’ and faculty's perception towards online learning. In addition, the faculty's competencies to adopt computer-based teaching make this method of teaching and learning more challenging. ^11^

On the 2 ^nd^ March 2020, KSA announced its first patient infected by Coronavirus. As a result, on 8 ^th^ March 2020, KSA suspended all schooling, including universities and other educational institutes, until further notice as an effort to control the virus outbreak. ^12^ In compliance with the KSA’s Ministry of Education’s ordinance regarding the suspension of educational activities, Imam Abdulrahman Bin Faisal University (IAU) and other universities within the region ceased all on-going events, hence, lectures and clinical sessions, and laboratories started to facilitate its students through virtual, online channels such as ZOOM and Microsoft’s Teams application.

Previous research has shown that the acceptance of online learning was deemed to be increasing amongst those students who have experienced it because students understand it as a more flexible way to learn that is less time consuming and allows students to learn in the comfort of their home. ^13^ Although there is evidence which suggests that the students are also missing the interpersonal environment which is only present in traditional learning when they meet teachers face-to-face. This research also demonstrates that they have to be more independent learners as there is limited tutor availability. ^14^^,^^15^ The situation we are currently facing concerning the pandemic and forced conversion to online learning for many institutions has created the need for further research into the student experience to ensure the quality of education has not been compromised. The objective of this research was to explore students’ perception of the implementation of online teaching and learning support. We identify the strengths and areas of improvements in online teaching and learning in higher education institutions in KSA. The aim is to gain a more rounded and detailed understanding of students' experiences of remote learning and the support they have received from their institutions during the COVID 19 lockdown, their satisfaction with the provided facilities as well as their continued engagement with online learning. Moreover, the findings from our study provide academicians the opportunity to further enhance the quality of pre-COVID-19 traditional face-to-face teaching methods as we look to retain the benefits from better understanding the virtual learning environment. In addition, identifying the strengths and weaknesses could lead to improvements for e-learning based on students' insights into their learning experiences.

## Methods

### Study design

A cross-sectional online-based study was conducted from May 2020 to June 2020 wherein a pre-validated questionnaire was generated through QuestionPro (advanced version). This questionnaire was sent via an online survey to all students of universities (IAU and SEU) in KSA. All students enrolled in the academic year 2019-2020 were eligible to participate in this study. Students’ email IDs were taken from the registrar’s office of the academic institutions to communicate details of the study to students. Students’ participation was voluntary, and a statement was incorporated in the survey form for participants to give their consent before they started the survey.

### Participants

The selection of student participants for the survey was done by purposive sampling. The selection process was designed to maximize the representation of diversity of the participants in terms of subject specialties (Dentistry, Medicine, Public Health), gender, and age to portray the wide spectrum of student backgrounds from the different health science colleges and universities in KSA. For the students to participate and to be included in the study they had to be currently enrolled in a KSA college or university and belonging to any specified discipline.

Moreover, consent to participate was given by the completion of the survey. A total of 350 students commenced the survey and 20% of them left the survey incomplete (did not reach the main questions). Therefore 281(80%) students who completed the survey were included in the final analysis of the study.

### Ethics

As this study involves human subjects, ethical approval is required under the bylaws of the Helsinki declaration. The institutional review board (IRB) in Imam Abdulrahman Bin Faisal University (IAU) is accredited by the Association for the Accreditation of Human Research Protection Programs (AAHRPP), which operates under the laws and regulations for human research in Saudi Arabia. For this study, the ethical approval was applied through the IAU Converis Management System, which is an electronic system that allows and facilitates research applications for members of IAU. The ethical approval for the study was obtained from the Institutional Review Board (IRB) at IAU (EA-202051).

### Questionnaire

The questionnaire was extracted and modified from a previously published study on distance-based education. ^16^ Modified questionnaires were pilot tested on 20 students belonging to both institutes (10 from each, traditional (IAU) vs online (SEU)) for reliability and validity. The questionnaire was divided into three sections, Demographic Information (consisting of 11 questions), Teaching and Learning Process (consisting of 9 questions with a forced 4-point Likert scale response), and Students Technical Support (consisting of 7 questions with 4-point Likert scale response). Cronbach alpha value for the Teaching and Learning section was obtained as 0.69, and for technical support, it was 0.76, which indicated acceptable internal consistency. Section 1 of the questionnaire covered the demographical characteristics of participants such as age, gender, institution’s name, department, Grade Point Average (GPA), parents’ education, and profession. Section 2 was based on the participants’ experience with the online learning responses amid COVID-19 and Section 3 consists of statements about technical support provided to the students in the case of any issue arising during lectures or examinations. There were two additional open-ended questions included to ask students about their future preferences and suggestions to further improve the quality of online learning.

### Statistical analysis

Statistical Package of Social Sciences (SPSS version 23, IBM USA) was used for data entry and analysis. For descriptive statistics, frequency distribution, percentages, averages, standard deviation, and bar graphs were created. For inferential statistics, two independent samples t-test was used to compare average scores with the questions that had two categories. Chi-square/Fisher's Exact Test was used for comparing variables with two or more categories. To handle the missing values, we assign discreet values (codes) during analysis in SPSS. The level of significance was set as 0.05 level of significance.

## Results

The number of students who participated in the study and completed the survey forms was 281. The average age was 23.1±4.5 years. About 68% of the participants were females (n = 188) while 32% (n = 88) were males. The distribution of students according to their institution and their academic level is presented in Table 1. There were 128 (45.6%) students who stated that they had a separate study room, 136 (48.4%) replied that they did not have a separate study room and 17 (6%) did not reply to the question. Analysis of the questions related to parents' qualifications revealed that the fathers were more qualified than the mothers. Table 1 also shows the qualification levels of the parents.

**Table 1.** Categorization of participating students according to demographics.

Institution	Frequency	Percentage
Imam Abdulrahman Bin Faisal University	155	55.2
Saudi Electronic University	69	24.6
King Abdulaziz University	1	0.4
Prince Sultan University	18	6.4
Others	38	13.6
**Academic year**		
2 ^nd^	73	26.0
3 ^rd^	84	29.9
4 ^th^	68	24.2
5 ^th^	18	6.4
6 ^th^	20	7.1
Master	1	0.4
Other	17	6.1
**Fathers’ qualification**		
Undergraduate	62	23.7
Graduate	119	45.4
Post-Graduate	49	18.7
Other	32	12.2
**Mothers’ qualification**		
Undergraduate	77	29.3
Graduate	109	41.4
Post-Graduate	39	14.8
Other	38	14.4

The questionnaire had nine questions which were asked to assess the students' satisfaction with the online teaching and learning process. It was found that students were very satisfied with the faculty’s prompt response in a variety of ways (mean score 3.03): their commitment in delivering lectures in terms of punctuality, preparation (mean score 3) and with providing online materials which helped them to better understand the course or topics (mean score 2.93) ( Table 2). Statistical significance was tested by comparing the response scores with demographic variables. Fathers’ education was found to have a statistically significant relation with questions “faculty is fully committed to the deliver lectures” and “faculty communication and provided online material” (p = 0.04 and 0.018, respectively). Mothers’ education had a significant association with the questions “faculty supported and responded quickly” and “during online lectures, faculty give break or extra time” (p = 0.042 and 0.026, respectively). In terms of the learning environment, the students who had a separate study room had significantly higher average scores for each question except for question 4 (“Feedback to student assignments is provided helpfully”) (p = 0.12). Similarly, students’ study year level had a significant association with their responses to statements “Faculty is promptly responding (Q1)”, “Faculty is fully committed (Q2)” and “Faculty supported(Q3)” (p = 0.035, 0.018, and 0.009, respectively). Specifically, senior students (final year students) showed higher satisfaction than junior students (foundation year, year 1 or 2 students).

**Table 2.** Students' satisfaction score with online teaching and learning process.

No.	Questions	Range	Mean	SD
Q1	Faculty is promptly responding to my queries regarding the course in a variety of ways. (sending lecture videos, lecture material, emails, WhatsApp message)	1-4	3.03	0.72
Q2	Faculty is fully committed to the deliver lectures (punctual, well-prepared in a manner that blackboard user, Confident, Zoom Familiar)	1-4	3.0	0.72
Q3	Faculty supported and responded quickly just like physical lecturing or face to face teaching	1-4	2.77	0.88
Q4	Feedback to student assignments is provided helpfully, Clearly and Timely manner)	1-4	2.58	0.96
Q5	Virtual Lectures conducted are consistent with the course learning outcomes provided at the start of the semester (Before Coronavirus lock-down crisis)	1-4	2.92	0.78
Q6	Courses are re-designed to minimize the load.	1-4	2.4	0.96
Q7	During online Lectures, faculty give break or extra time to think and understand the lectures thoroughly.	1-4	2.69	0.84
Q8	Faculty communication and provided online material is well enough to understand the topics.	1-4	2.93	0.94
Q9	Do you feel any stress after taking first online lecture/exam	1-4	2.66	0.86

Evaluation of questions about students’ satisfaction towards the technical support provided during the teaching process is presented in Table 3. Demographic questions were analyzed with questions tabulated in the table “How do you rate the IT services” and the question “Was the lecture time provided to cover the specific topic enough” were found to correlate with the students’ study year (p = 0.033) which showed a significant increase in averages due to increasing in academic level. Question 5 (“Was the lecture time provided to cover the specific topic enough”) also had a significant association with fathers' and mothers' education levels (p = 0.036 and 0.035, respectively). The question "do you have a separate study room?" had a significant relationship with the questions “how do you rate the internet connection”, “how do you rate the IT services” and “overall, I am satisfied with the e-learning”. Those who had separate study rooms had a significantly high average score compared to their counterparts (p = 0.019, 0.002, and 0.0001, respectively). Overall, the students agreed (62%) that they are satisfied overall with the e-learning process.

**Table 3.** Students' satisfaction score regarding technical support during the online teaching process.

No.	Questions	Range	Mean	SD
Q1	How do you rate the internet connection provided in your area/home	1-4	2.66	0.86
Q2	A self-guided instruction regarding online lecturing was provided ahead of time.	1-4	2.81	0.71
Q3	How do you rate the IT services provided to support during the online lecturing/quiz/exam	1-4	2.77	0.78
Q4	Did you satisfy the IT services provided in case of technical issue happened during the exam or lecture?	1-4	2.73	0.79
Q5	Was the lecture time provided to cover the specific topic enough?	1-4	2.83	0.80
Q6	Sufficient library resources are made available to the students.	1-4	2.96	0.75
Q7	Overall, I am satisfied with the e-learning (lectures and examination) until this situation of COVID-19 is over completely.	1-4	2.75	0.94

**Figure 1.**
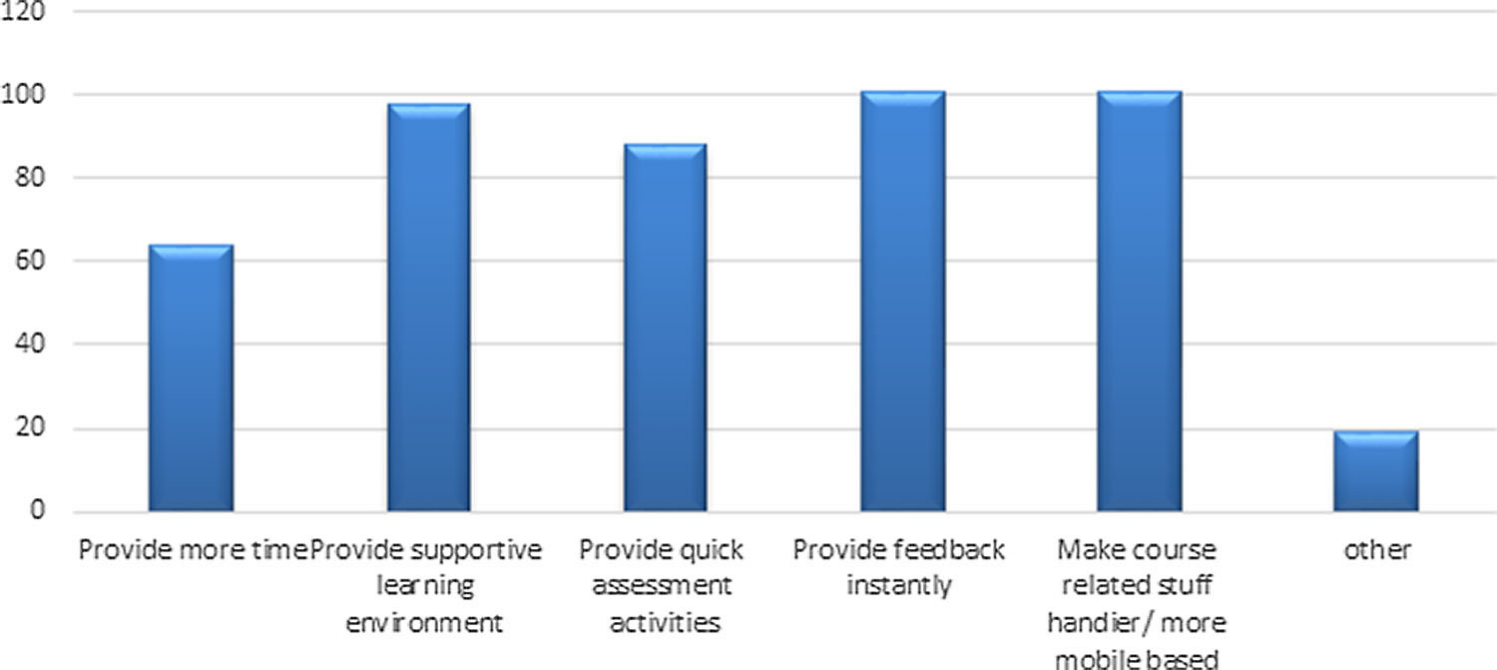
Students suggestions for the instructor to do differently to help them to learn more.

**Figure 2.**
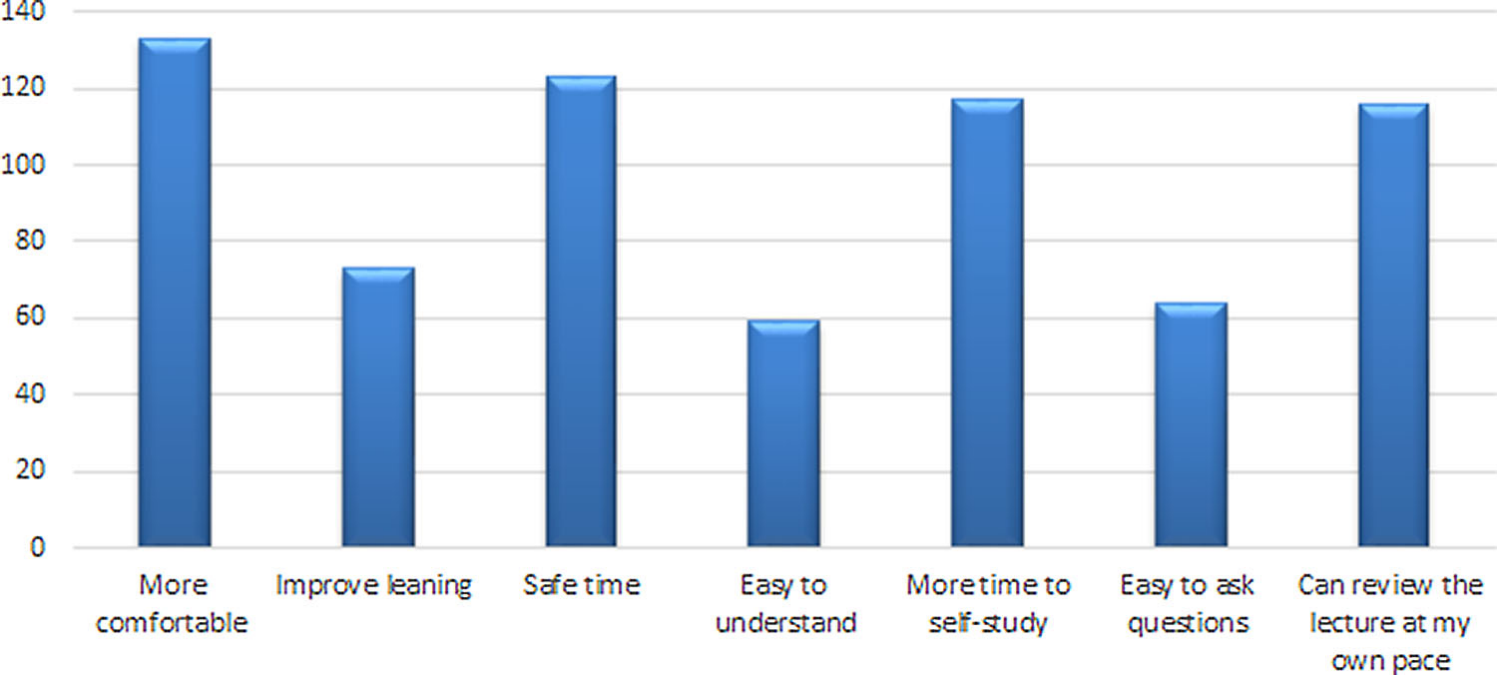
Responses of students for recommending E-learning in the future.

**Figure 3.**
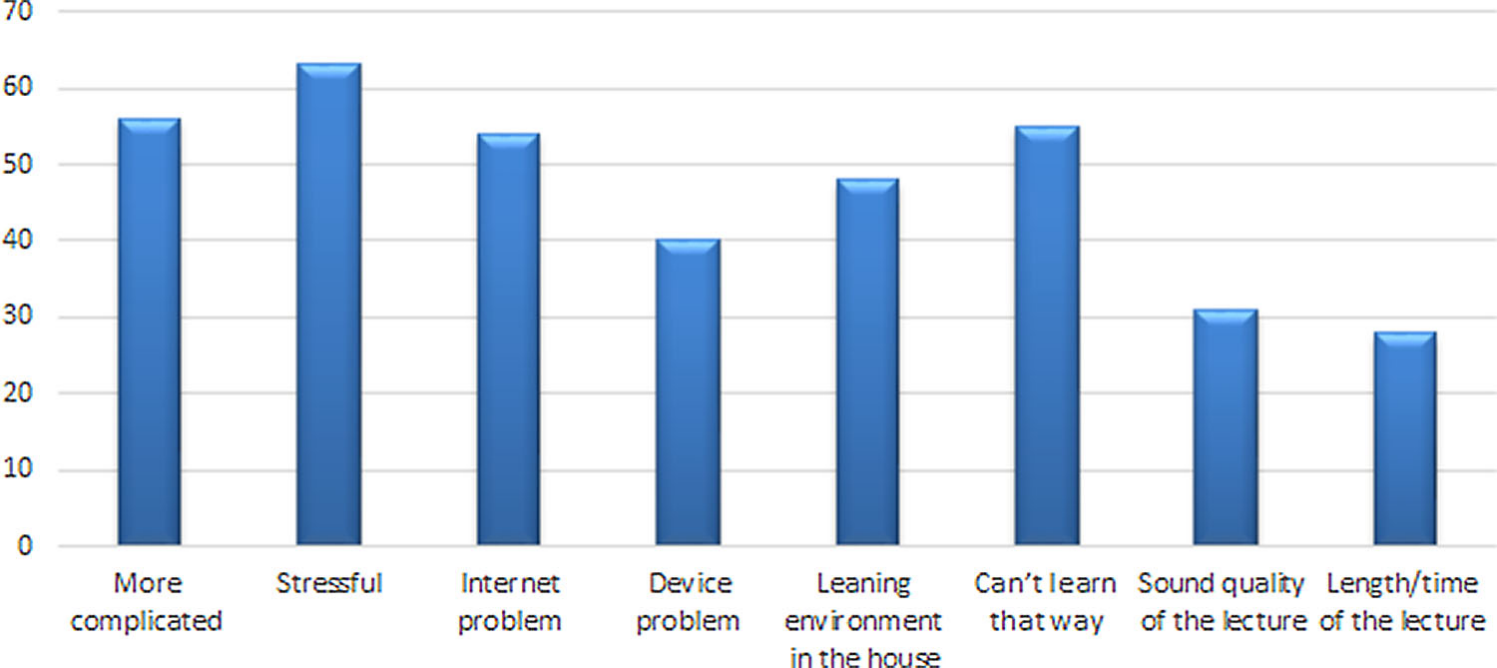
Response of students for not recommending E-learning in future.

Evaluation of students’ responses about what an instructor can do differently to help them learn, showed that "provide feedback instantly" and "make course-related stuff handier/more mobile base" were the options with the highest responses ( Figure 1). Furthermore, 163 (63.2%) had agreed that they would recommend E-learning in the future while 95 (36.8%) had disagreed that they would recommend this for future use. The most common reason for recommending E-learning in the future was students feeling more comfortable ( Figure 2). Conversely, the stress of using the online system was the most common reason for not recommending E-learning in the future ( Figure 3).

Students’ type of institution (online vs traditional) was analyzed with the question “would you recommend this e-learning in the future”. The comparison was provided that a significantly high proportion of students (87.3%) who belonged to E-learning institutions had agreed that they would recommend E-learning in the future compared to 57.6% of traditional institution students (p = 0.0001). Furthermore, the institution type was analyzed with questions relating to technical support provided during lectures, and average scores and p-values were tabulated in Table 4. We found four questions where the difference between the satisfaction of students belonging to traditional institutes was signficantly different than those belonging to blended online institutes (SEU). Selfguided instruction, duration of lecture time and overall satisfaction was scored high by the students of e-learning institutes (p-values = 0.001, 0.047, 0.0001 respectively).

**Table 4.** Comparsions of average scores with standard deviation between the type of institution.

No.	Questions	Institution Type		P-value
		Traditional	E-learning	
Q1	How do you rate the internet connection provided in your area/home	2.61 (0.88)	2.78 (0.79)	0.184
Q2	A self-guided instruction regarding online lecturing was provided ahead of time.	2.75 (0.72)	3.08 (0.6)	0.001 ^*^
Q3	How do you rate the IT services provided to support during the online lecturing/quiz/exam	2.74 (0.77)	2.92 (0.82)	0.115
Q4	Did you satisfy the IT services provided in case of technical issue happened during the exam or lecture?	2.67 (0.79)	2.92 (0.79)	0.045 ^*^
Q5	Was the lecture time provided to cover the specific topic enough?	2.82 (0.81)	3.05 (0.66)	0.047 ^*^
Q6	Sufficient library resources are made available to the students.	2.98 (0.74)	2.92 (0.7)	0.556
Q7	Overall, I am satisfied with the e-learning (lectures and examination) until this situation of COVID-19 is over completely.	2.66 (0.91)	3.24 (0.78)	0.0001 ^*^

* Statistically significant with 0.05 level of significance.

## Discussion

Online learning has revolutionized teaching and learning in higher education institutions during the recent pandemic. ^17^ Most internationally ranked university students are far more confident in terms of asynchronous learning opportunities through the instructional use of e-mail, chat rooms, bulletin boards, online lectures through Blackboard Ultra or Zoom as well as online assessments. ^18^^–^^19^ The technologies are transforming education from the perspectives of learners, instructors, IT managers, and administrators. Therefore, examining perceptions of learners’ experiences is a widely used strategy, which often influences behavior. ^20^ It is important to study students’ perceptions of online course quality for institutions, administrators, faculty, and students themselves in order for the online learning environment to be successful.

Although virtual teaching is not very popular or common among many Saudi institutions, the COVID-19 pandemic presents an extraordinary situation worldwide, which became even worse from time to time during the end of the academic year where there were a lot of uncertainties regarding the teaching of course material, course completion, examination, and assessments. The present study aims to evaluate health sciences students' perceptions about online learning in Saudi higher education, which replaced traditional face-to-face teaching due to the COVID-19 pandemic across the globe.

In the present study, Saudi students’ responses highlighted areas of good practice and areas for further development and/or improvement. The areas of good practice found in this study were the faculty’s prompt responses to students’ queries related to teaching material and/or assessments, faculty commitments and preparation to provide online lectures, and maintaining contact with students’ during the lockdown crisis. However, students were not satisfied with the course design which suggests that there is a need to modify each course teaching, learning, and assessment methodology that was originally designed to be delivered face-to-face. However, since extensive online teaching was introduced without any prior notice to students and planning from the teaching staff, this was an expected finding. Every institution needed to be well prepared to design the module specification more flexibly and make adjustments in such a way that students adapt to both face-to-face and online teaching, learning, and assessment changes without any stress which may influence performance in their courses.

The areas of further development that respondents mentioned were delayed feedback from the faculty and a lack of group work or team-based learning. ^21^ However, there were innate challenges with the implementation of this with differing class sizes, accessibility, disability issues, and facilities. It also warrants some adjustments in the learning outcomes but may raise issues where time is constrained. Therefore, continuous informal feedback (talking to students or their tutors), students' written feedback (module feedback surveys) or critically reviewing their performance in course assessments and through direct feedback are more beneficial tools and key to education to enhance learning development.

In the present study, parents’ education is also found to be a significant confounder for students’ satisfaction, which may suggest that educated parents may tend to be more concerned and provide more support to facilitate their children’s education. This is consistent with a previous study that stated that parenting behavior influences children’s education. ^22^ Our study found that those who have a separate study room are more in favor of online teaching. ^22^ The home study environment is believed to have a positive influence on students’ learning ^13^ - and the present study suggested that students who have a spare room for their studies are more satisfied with their learning experience.

It could be deemed that the responses provided to several of the questions/statements indicated that the students were positively disposed towards some aspects of online learning: namely, communication and faculty provided online material which helped students to understand the topics and contributed to their satisfaction with the technical support provided to them during lectures or examinations. Additionally, 19% found it is more comfortable and 18% thought that online teaching was less time consuming. Conversely, 17% found it took more time to study while more than 50% agreed that they would recommend it in the future but with some concerns (e.g. more complicated, stressful, internet problem in the area or cannot learn this way).

Technical support provided during exams and lectures was also appreciated by the students. The overall satisfaction with online teaching demonstrated by students was 68%, indicating their agreement towards the continuation of E-learning. Providing feedback instantly, open discussion sessions, and improving availability of resources were the highest-rated questions (21%, 21%, 20% respectively).

This study also explored students’ opinions on how the faculty performed differently to support students' learning and >60% of the population suggested an effective learning environment, regular assessment activities, and instant feedback would be beneficial to monitor students' learning which is in line with the findings of a previous study. ^23^ Moreover, technology-enhanced learning may make it easier for students to follow their course content electronically at their own pace. ^24^ Various studies highlighted technology-enhanced learning and teaching as a significant revolution in all educational settings. Literature also suggests smartphones as a new paradigm in higher education for overcoming obstacles ^25^ and educational providers may also consider using apps such as Menti, Kahoot, Formative, Socrative, and Turning Point to assess learning of larger groups. The most unique benefit is that there are many ways through which learning can be more effective thus further enhance the quality of online teaching practice in higher education.

Furthermore, the study highlighted some factors pertaining to why participants may favor virtual learning. More than 50% of participants stated that it is more comfortable, time-saving, provides time for students to do self-study, and of course since the lectures are recorded students can review them as many times as desired to get a better understanding, thus improving students’ engagement. This does however raise the question of how actively all students are engaging in the online sessions. Meeting and communication tools such as Collaborate, Zoom, and Microsoft Teams have features to promote active learning through audience participation, offering the possibility of enhancing engagement through student participation. However, it has been suggested that involvement in an online learning session is not necessarily the same as engagement, and the more vocal students are often those who are only engaging in learning at a surface level. ^26^ Students who have a lower profile may well still be learning and benefitting from the experience ^27^, even if not contributing to the group activity. Emphasizing participation and rewarding this behavior does not recognize differences in learning processes, where students may be exhibiting a choice to learn in silence. ^28^ Many students prefer to remain anonymous, preserving their privacy and perceived safety, and find their silence empowering. ^29^ Furthermore, enforced participation can lead to disengagement. ^30^ Encouraging rather than rewarding anonymous participation may, therefore, improve engagement. However, it does beg the question of whether or not students should be judged on their level of engagement and participation and whether participation in these online sessions is a sensible way to assess if they are helping the students achieve the learning outcomes for the session. Among those who chose ‘not recommended’ (36%), they found online teaching ‘stressful’ (20%), ‘complicated’ (15%) and ‘cannot learn this way’ (14%) was the highest rated response, and therefore it is essential to identify factors of stress that negatively influence the students’ learning which may guide the faculty to make certain modifications to make this mode of learning less stressful and more interesting for students.

Overall, the method of using a questionnaire in this study was successful in exploring students' perceptions of their learning experience. It also explored their opinion about the most effective way to maximize their learning. Based on the findings, the results guided us to consider the modification of a range of areas related to students’ learning, including assessment and feedback mechanism, approaches to teaching, and improving students’ engagement and professional development, which would enhance the learning experience and cater for the diversity of students within the program.

This is a small study but will feed into the curriculum development of the different courses across a range of programs. There is a growing appreciation that a student’s attitude can have an impact on a student’s achievement, thus consideration of incorporating these findings when designing a curriculum will be highly beneficial.

Furthermore, this study will also benefit the students who are stakeholders in higher education institutions and who are considered to be one of the vital contributors to this educational inquiry. It is hoped that this study will benefit the students who participated as their institutions make changes to courses. The findings from this exploration of students’ attitudes can support the development of a positive online learning experience, as well as guide the faculty members in designing their course specifications and program curricula to best utilize the virtual learning environment and systems. It has shown that if students and faculty members are treated not simply as stakeholders but as partners, teaching and learning strategies will be enhanced further in relation to online education. ^31^

## Study limitations

There are vast limitations to this study. The number of participants was around 20% less than expected. This may be due to multiple factors including the more stressful and unprecedented situation students are experiencing during the pandemic. The questionnaire was kept short and took no longer than 5 minutes to complete. We know the students surveyed were in the last phase of the final examination and all other educational activities were concluded so it was more difficult to reach them.

Another limitation is related to the questionnaire used in this study. Although questionnaires and interviews are normally deemed to provide qualitative information, the use of a Likert-scale would allow the conversion of questionnaire data to numerical data. ^32^^–^^34^ This scale is often a 5-point ordinal scale that respondents use to rate the degree to which they agree or disagree with a statement on a symmetric agree-disagree scale. This allows responses to be ranked, but strictly speaking, the distances between the different responses is not measurable or necessarily equal. Nevertheless, this is a technique frequently used in educational research to provide quantifiable data. ^35^

The use of closed questions in the present study questionnaire that were incorporated in this inquiry is generally considered an effective and efficient technique. However, such questions could have risked being suggestive, allowing for misconceptions and missing crucial, pertinent, facts that the respondent would like to convey. ^36^

Moreover, the questionnaire used in this study was in the English language and therefore may be considered as one of the most important limitations as the participants’ native language is Arabic. They may, therefore, respond more accurately if the questionnaire was in the Arabic language or a bilingual version to ease the comprehension of the respondents.

Overall, the majority of the students, 176 (62%), were satisfied with the online learning process which gave the same perception as presented by a recent study in Poland among medical students. ^37^ We have learnt from this study that if it were to be conducted again, some modifications would need to be performed in the research design and tools to make this study more conclusive. For instance, collecting data from all students across the different programs in Saudi Arabia or to include faculty and other teaching staff to enhance the quality of online teaching and learning.

The findings also emphasize the role of faculty on influencing students’ experience by effective communication which also has greater impact on students’ learning. ^38^ This also suggests that increasing the use of technology and faculty training will improve their teaching practice using technology. ^38^ However, this needs a careful evaluation to identify areas of improvement in teaching, to have a positive effect on educational outcomes. In addition, further studies are needed to explore how the quantity and quality of technology use affect student learning outcomes over time and what type of technology may be more popular among students as well as faculty. ^39^

Furthermore, long term data may also guide higher education institutions to make informed judgments on faculty’s strengths and concerns about using technological tools for teaching.

## Conclusion

The findings were intended to inform teaching practice and guide the faculty and staff to develop a mechanism that allows the students to share their learning experiences. Analyzing students’ perception across most of the health sciences programs in Saudi higher education institutions will provide evidence to support the faculties which are looking to increase students’ satisfaction and enhance the quality of the learning experience, which in turn should intensify students’ engagement. ^40^ Sixty two (62%) percent of students were found to be satisfied with the e-learning process. Incorporating suitable survey practices will also guide the faculties to regularly review and modify the curriculum design and make both the module specifications and the teaching methods more learner or student-centered. ^41^

The compelling trend towards the utilization of electronic resources in the field of education and the development in technology is appealing and challenging. One way to help improve and develop students’ intuitive minds in this modernized technological environment is by utilizing electronic resources to actively indulge students to facilitate effective teaching and learning processes through E-learning. ^42^

Overall, this study has helped researchers and faculties to identify strengths and also areas of improvement in the E-learning process, including encouraging faculties to think deeply into the restructuring of course learning objectives and teaching techniques to engage students and improve the wider learning process. Thus, the findings of this study can serve as a tool for others to develop programs that facilitate the advancement of E-learning into an effective mode of teaching and learning to enhance the range of methods of educational delivery in Saudi Arabia. Particularly in response to the present situation of the so-called “new normal” that is being caused by COVID-19 pandemic.

## Data availability

### Underlying data

Harvard Dataverse V2: Perception on Online Teaching and Learning Among Health Sciences Students in Higher Education Institutions during the COVID-19 Lockdown – Ways to Improve Teaching and Learning in Saudi Colleges and Universities, https://doi.org/10.7910/DVN/3KJGBD V2; License CC0. ^43^

This project contains the following extended data:

Farooqi, Faraz (2021), “Perception on Online Teaching and Learning Among Health Sciences Students in Higher Education Institutions during the COVID-19 Lockdown – Ways to Improve Teaching and Learning in Saudi Colleges and Universities”, Mendeley Data, V1, https://dx.doi.org/10.17632/r9vkdj8pw6.1. ^44^
