# Supplementary material for: Perception on Online Teaching and Learning Among Health Sciences Students in Higher Education Institutions during the COVID-19 Lockdown – Ways to Improve Teaching and Learning in Saudi Colleges and Universities
Source: F1000Res. 2021 Dec 7;10:177. Originally published 2021 Mar 4. [Version 2] doi: 10.12688/f1000research.28178.2 (PMC7993406; doi:10.12688/f1000research.28178.2)
Supplement: Peer review history

# Peer review: Reviewer response for version 1

**DOI:** 10.5256/f1000research.31170.r80945

**Published:** 2021-03-11

**Authors:** Imran Alam Moheet

**Affiliations:** 1 Baqai Dental College, Baqai Medical University, Karachi, Pakistan

- Overall, the authors have done a great job. It is a very important topic under the current circumstances.
- The references need to be proofread again as all of them are not in the same format.
- The conclusion is a bit long, The first two paragraphs of the conclusion seem to be part of the discussion; if possible rephrase or move it to the discussion.
- References should not be used in the conclusion ****unless the journal allows it.
- The reason for referencing the article by Farooq *et al. *(2020 ^1^) is that it contains relevant information regarding e-learning during covid-19 and it is very current (2020) and the authors should use it as a reference in their discussion instead of articles that are a decade old.

Is the work clearly and accurately presented and does it cite the current literature?

Yes

If applicable, is the statistical analysis and its interpretation appropriate?

I cannot comment. A qualified statistician is required.

Are all the source data underlying the results available to ensure full reproducibility?

Yes

Is the study design appropriate and is the work technically sound?

Yes

Are the conclusions drawn adequately supported by the results?

Yes

Are sufficient details of methods and analysis provided to allow replication by others?

Yes

Reviewer Expertise:

Dental Education, Tertiary education, Dental materials, biomaterials,

I confirm that I have read this submission and believe that I have an appropriate level of expertise to confirm that it is of an acceptable scientific standard.

## References

[1] : COVID-19 outbreak, disruption of dental education, and the role of teledentistry. *Pak J Med Sci* .36(7) : 10.12669/pjms.36.7.3125 1726-1731 10.12669/pjms.36.7.3125 33235605PMC7674864

# Response

**Published:** 2021-04-06

**Authors:** Faraz Farooqi

**Affiliations:** Imam Abdulrahman Bin Faisal University, Saudi Arabia

Dear Dr. Imran,

 Thank you for your time and efforts to review this article. The mentioned reference is already cited on position 42 in the discussion. Other valuable suggestions were also incorporated in the text.

 Thank you and best regards.

# Peer review: Reviewer response for version 1

**DOI:** 10.5256/f1000research.31170.r80947

**Published:** 2021-03-05

**Authors:** Muhammad Naveed Anwar

**Affiliations:** 1 Department of Computer and Information Sciences, Northumbria University, Newcastle-upon-Tyne, UK

- It is an interesting piece of work especially in the current scenario. It is good to see that the survey questionnaire and data for this study is accessible to other researchers for further work.
- The title of paper mentions “Perception on online teaching and learning among **Health Sciences Students.**..” but this research work does not discuss it. It can be removed from the title and from the text if it is not discussed.
- It would be good to have chi-squared values but they are not provided.
- The “Results” heading on the very first page needs +/-4.5 only 4.5 is not clear.
- This paper needs proof reading.
- There are some very small paragraphs; they need to be joined with other paragraphs or extended.

Is the work clearly and accurately presented and does it cite the current literature?

Yes

If applicable, is the statistical analysis and its interpretation appropriate?

Yes

Are all the source data underlying the results available to ensure full reproducibility?

Yes

Is the study design appropriate and is the work technically sound?

Yes

Are the conclusions drawn adequately supported by the results?

Yes

Are sufficient details of methods and analysis provided to allow replication by others?

Yes

Reviewer Expertise:

Data mining, data analysis, data science, big data, statistical analysis, online teaching and learning, online businesses, digital businesses.

I confirm that I have read this submission and believe that I have an appropriate level of expertise to confirm that it is of an acceptable scientific standard.

# Response

**Published:** 2021-04-06

**Authors:** Faraz Farooqi

**Affiliations:** Imam Abdulrahman Bin Faisal University, Saudi Arabia

Dear Dr. Naveed,

 Thank you for your valuable suggestion to improve the study. We have modified the article as per your comments. Kindly note that p-values are mentioned within the text now and health sciences students is now mentioned as well.

 Thank you again for your time as well.

 Best regards.
